# Modeling the global distribution of Culicoides imicola: an Ensemble approach

**DOI:** 10.1038/s41598-019-50765-1

**Published:** 2019-10-02

**Authors:** Samson Leta, Eyerusalem Fetene, Tesfaye Mulatu, Kebede Amenu, Megarsa Bedasa Jaleta, Tariku Jibat Beyene, Haileleul Negussie, Crawford W. Revie

**Affiliations:** 1 Addis Ababa University, College of Veterinary Medicine and Agriculture, P. O. Box 34, Bishoftu, Ethiopia; 2 National Animal Health Diagnostic and Investigation Centre (NAHDIC), P. O. Box 04, Sebeta, Ethiopia; 3 Center for Outcome Research and Epidemiology, Kansas State University, Manhattan, Kansas USA; 4 Department of Computing and Information Sciences, University of Strathclyde, Livingstone Tower (14.01), 26 Richmond Street, Glasgow, G1 1XQ Scotland UK

**Keywords:** Ecological modelling, Ecological epidemiology

## Abstract

*Culicoides imicola* is a midge species serving as vector for a number of viral diseases of livestock, including Bluetongue, and African Horse Sickness. C. imicola is also known to transmit Schmallenberg virus experimentally. Environmental and demographic factors may impose rapid changes on the global distribution of *C*. *imicola* and aid introduction into new areas. The aim of this study is to predict the global distribution of *C*. *imicola* using an ensemble modeling approach by combining climatic, livestock distribution and land cover covariates, together with a comprehensive global dataset of geo-positioned occurrence points for *C*. *imicola*. Thirty individual models were generated by ‘biomod2’, with 21 models scoring a true skill statistic (TSS) >0.8. These 21 models incorporated weighted runs from eight of ten algorithms and were used to create a final ensemble model. The ensemble model performed very well (TSS = 0.898 and ROC = 0.991) and indicated high environmental suitability for *C*. *imicola* in the tropics and subtropics. The habitat suitability for *C*. *imicola* spans from South Africa to southern Europe and from southern USA to southern China. The distribution of *C*. *imicola* is mainly constrained by climatic factors. In the ensemble model, mean annual minimum temperature had the highest overall contribution (42.9%), followed by mean annual maximum temperature (21.1%), solar radiation (13.6%), annual precipitation (11%), livestock distribution (6.2%), vapor pressure (3.4%), wind speed (0.8%), and land cover (0.1%). The present study provides the most up-to-date predictive maps of the potential distributions of *C*. *imicola* and should be of great value for decision making at global and regional scales.

## Introduction

Globally, the incidence of vectors and vector-borne diseases of livestock and humans is increasing at an alarming rate associated with changes related to factors such as climate, environment, high human mobility, unplanned urbanization and agricultural intensification^1,2^. Mosquitoes, ticks, sandflies, tsetse flies and biting midges are the common vectors transmitting agents of vector-borne diseases that cause significant production and productivity losses in livestock systems^3^. Among biting midges (Culicoides species), *Culicoides imicola* is one of the most widespread in the world^4^. *C*. *imicola* transmits the agents of a number of viral diseases of veterinary importance, including Bluetongue^5,6^, and African Horse Sickness (AHS)^7,8^. *C*. *imicola* is also known to transmit Schmallenberg virus experimentally^9^.

Bluetongue is a devastating viral disease of ruminants found to be historically enzootic in tropical regions of the world; however, since 1998 the virus has spread across southern European countries. *The expansion is believed to be facilitated by northward distribution of the infected Culicoides species mainly C. imicola and availability of competent and efficient vectors such as C. obsoletus and C. pulicaris*^10–12^. AHS is an infectious disease considered to be the most lethal viral disease of equines, especially horses^7,13^ and is endemic in sub-Saharan Africa^7^. Schmallenberg virus is a recently emerged virus, identified in North Rhine-Westphalia, Germany, during the summer of 2011^14^, which has since spread across Europe inflicting congenital deformities in the offspring of infected adult ruminants^15^. The recent emergence of two Culicoides-borne diseases (Bluetongue and Schmallenberg) in Europe has raised concerns around the potential introduction and further spread of AHS virus into temperate parts of the world^13^.

As stated above, *C*. *imicola* is a cosmopolitan midge species and has been reported from various geographic areas of the world spanning in its distribution from South Africa to southern Europe and from western Africa to southern China^4^. The recent northward expansion of *C*. *imicola* and unprecedented outbreaks of Bluetongue and Schmallenberg viruses in southern Europe have been a major research and surveillance focus. Knowledge of the potential geographical distribution of this species is important to guide surveillance of *C*. *imicola* and the diseases it transmits. Considerable numbers of studies have mapped the national or regional distributions of *C*. *imicola* using a range of modelling techniques. For example, maps outlining the distribution and ecological niche of *C*. *imicola* have been developed for Spain, Portugal, Morocco, Italy, the Mediterranean basin and South Africa^16–20^.

Modeling the spatial and temporal distribution of vector species can help in the assessment and management of the associated health risks^21^. However, the global distribution models of *C*. *imicola* so far developed^4^ have been based on coarse-scale images, specifically using CLIMEX at 10’ spatial resolution. Moreover, the majority of previous studies focused on assessing the distribution and survival of this species by considering only a few climatic covariates and adopted a single-model forecasting technique. In contrast, ensemble modeling techniques combine a variety of modeling techniques to better predict the global distribution of *C*. *imicola* and also characterize the respective contributions of each variable. Ensemble models are meta-algorithms that combine several modeling techniques into a single predictive model in order to decrease variance and bias, and improve prediction^22,23^. The present study was initiated based on the proposition that an ensemble modeling technique could be used to predict the global distribution of *C*. *imicola*, and ultimately provide better scientific evidence with regard to the potential global distribution of the vector.

## Materials and Methods

In this study a probabilistic global habitat suitability model for *C*. *imicola* was developed using an ensemble modeling technique^23^. The modeling was implemented using the R package biomod2^23^. The developed models combine climatic, livestock distribution and land cover covariates to predict the global distribution of *C*. *imicola* and to characterize the respective contribution of various factors. The following sets of input data were used in order to make accurate predictions of the distribution of this vector: (i) environmental and livestock distribution data which includes climatic, land cover and livestock population data, and (ii) a globally comprehensive dataset of geo-positioned occurrence points for *C*. *imicola*. Details regarding the specific attributes of the model and data generation are outlined below.

### Environmental and livestock distribution data

#### Climatic data

The survival of a given vector/species and thereby their geographical distribution is influenced by climatic and other environmental factors. As is the case for many other insects, the distribution of *Culicoides* is governed by climate factors^4,24–27^. Climatic factors, particularly temperature and rainfall can promote, enhance or even break critical parts of the life cycle for a given species. Solar radiation^28^, wind speed^29,30^, and water vapor pressure^31^ have also been reported to influence the presence of different insect species. To account for the impact of climatic factors on the distribution of *C*. *imicola*, data were downloaded from the WorldClim database (http://worldclim.org/). WorldClim version 2^32^ has average monthly climate data including minimum, mean, and maximum temperature as well as precipitation for 1970–2000. Solar radiation (kJ m^−2^ day^−1^), wind speed (m s^−1^) and water vapor pressure (kPa) are also available in version 2 of the Worldclim database. The database provides these climatic layers at different spatial resolutions, from 30 seconds (~1 km^2^) to 10 minutes (~18 km^2^); 2.5 arcminute resolution data (~5 km^2^) were used in this study.

#### Land cover data

To account for the impact of land usage on the distribution of *C*. *imicola*^27^, land cover data were downloaded from the European Space Agency’s GlobCover Portal (http://due.esrin.esa.int/page_globcover.php). In this study GlobCover v2009, released on 21^st^ December 2010, was used. This dataset is the most recent (2009) available and specifies 22 classes^33^, based on the Land Cover Classification System (LCCS) at a high resolution (300 m).

#### Livestock distribution data

*Culicoides* rely on livestock for their blood meal and according to Purse *et al*.^27^, local-scale abundance patterns of *Culicoides* are best explained using models that include data relating to potential hosts. To account for the impact of host distribution on the distribution and occurrence of *Culicoides*, livestock distribution data was downloaded from the website of FAO livestock systems (http://www.fao.org/livestock-systems/) and incorporated into the model. The livestock dataset contains peer-reviewed spatial distribution for cattle, sheep, goats, buffaloes, horses, pigs, chickens and ducks at a global extent with 5 minutes of arc (~10 km^2^) resolution for the year 2010^34^.

#### Culicoides occurrence data

The *C*. *imicola* occurrence records compiled and published by Leta *et al*.^35^ which contains information on the known global occurrences of *C*. *imicola* was used in the present study. The data consists of 2 589 (before technical validations) and 1 039 (after technical validations) geo-positioned occurrences of *C*. *imicola* spanning 50 countries worldwide. Geographical biases in the density of occurrence records were apparent in the data before technical validations, during technical validations; a 5 km spatial thinning was performed to overcome the geographical sampling bias. Thus, a more comprehensive global database of *C*. *imicola* occurrence consisting of 1 039 spatially thinned occurrence records used as occurrence records in the ensemble species distribution modeling of the present study.

#### GIS operation and variable selection

It was found that the various databases had different spatial resolutions; land cover data has 300 m^2^, the climatic data has 5 km^2^ and the livestock data has 10 km^2^ spatial resolutions. This necessitated rescaling. Accordingly, the databases were rescaled to 2.5 arcminute (~5 km^2^) resolution to match the WorldClim database spatial resolution. Multicollinearity among explanatory variables was checked using variance inflation factor (VIF) analysis^36^, with the “*vifstep”* command in the “*usdm”* package of R^37,38^. A stepwise selection routine was implemented to select a set of variables with sufficient low multicollinearity and only variables which had VIF values less than or equal to 10 were considered in the analysis^39^. However, according to Guisan and Zimmermann^40^, the selection of explanatory variables should also be based on the selection of conceptually meaningful variables. Thus, due to its eco-physiological importance^4,24,25^ average annual maximum temperature was kept in the model despite its high VIF value.

#### Modeling approach

The potential distribution of *C*. *imicola* was estimated based on ensemble species distribution modeling, executed using the biomod2 package in R. The package produces ensemble species distribution models using ten different methods: general linear models (GLM), general boosted models (GBM, also referred to as boosted regression trees), general additive models (GAM), classification tree analysis (CTA), artificial neural networks (ANN), surface range envelope (SRE), flexible discriminant analysis (FDA), multiple adaptive regression splines (MARS), random forests (RF), and maximum entropy (MAXENT)^23^. All ten modelling techniques require both absence and present records to determine the suitability range for the species under question. Explicit *C*. *imicola* absence records were not available, so pseudo-absence data were generated using the Surface Range Envelope (SRE) model. This technique forces pseudo-absences to be selected outside of the broadly defined environmental conditions suitable for the species. As such a surface range envelop model is first generated for the species of interest, after which the pseudo-absence data are defined as occurring outside of this envelop^23^.

To evaluate the performance of each model, the *C*. *imicola* occurrence records were split into two, with 80% of the data being used to train and 20% to test the models. The true skill statistic (TSS) and the area under the receiver operating characteristics (ROC) curve were used to assess the performance of the models^41,42^. Three evaluation runs were performed during the modeling, resulting in a total of 30 models (10 modelling methods × 3 folds), from which the average values of TSS and ROC were taken. TSS scores range from −1 to 1, where +1 indicates a perfect ability to distinguish actually suitable from unsuitable habitat, while values of zero or less indicate a performance no better than random^41^. For the ensemble modeling, only those models with a TSS value greater than 0.8 were considered^41,42^.

The models produced raster cells with values varying between 0 and 1000. The values indicate how close the climate and ecological conditions within in each cell are to the optimal conditions for the species in question; with higher values indicating higher suitability. As a rule of thumb, sites with suitability higher than 500 predict presence, while sites with suitability lower than 500 indicate absence. The estimated suitability value was divided by 1000 to convert the suitability value into a probability of occurrence. During model development the ‘build.clamping.mask’ was set to ‘TRUE’ to identify locations where predictions could be uncertain. Predictions could be uncertain if values of the variables extend outside the range used for calibrating the models. Models committee averaging, which gives both a prediction and a measure of uncertainty, was also developed during the ensemble modeling.

## Results

### Model performance and importance of environmental variables

**Figure 1:**
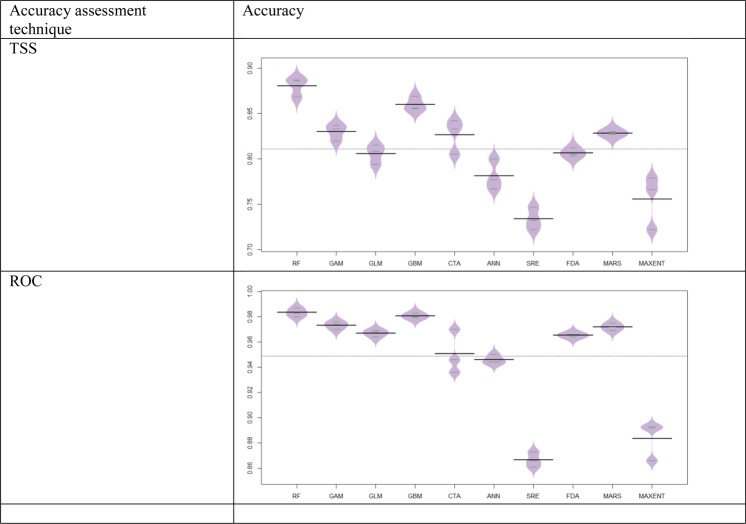
Beanplot illustrating performance in terms of TSS and ROC values over the 30 prediction models (10 algorithms × three runs). The lite horizontal lines indicate the overall averages.

The ensemble model performed very well (TSS = 0.898 and ROC = 0.991). Figure 1 illustrates the TSS and ROC scores of the 30 models. On average the most accurate technique was random forest, while the least accurate was surface range envelope. Among the 30 models, 24 had ROC > 0.90 (ROC_average_ = 0.95), considered as good accuracy based on the classification of Swets^43^. Of these, 21 had TSS > 0.8 (TSS_average_ = 0.81) or excellent accuracy based on the classification of Ben Rais Lasram *et al*.^44^. High-accuracy models (TSS > 0.8) were combined to form ensemble forecasting of *C*. *imicola*.

The distribution of *C*. *imicola* is mainly constrained by climatic factors. Both the individual and the ensemble models identified two temperature variables, namely mean annual minimum and mean annual maximum temperature, as significant determinants of the distribution of *C*. *imicola*. The variables contributions of individual models are provided in Table 1.

**Table 1:** Contribution (%) of each variable to the variability in the initial models.

Variables	RF	GAM	GLM	GBM	CTA	ANN	SRE	FDA	MARS	MAXENT	Overall relative contribution
Tmin	21	46	45	20	10	42	15	49	53	23	35.2
Tmax	7	23	26	12	13	18	17	28	24	18	19.9
Srad	17	15	18	11	20	13	16	10	9	11	13.8
Prec	11	9	8	12	13	8	9	11	9	18	10.2
Livestock	28	0	0	28	21	8	15	0	5	7	8.7
Vapr	14	4	1	17	20	11	16	2	1	10	8.5
Wind	3	2	2	1	3	1	8	1	0	12	3.0
Land cover	0	1	0	0	0	0	4	0	0	2	0.8

Key: Tmax = Mean annual maximum temperature (°C), Tmin = Mean annual minimum temperature (°C), Srad = Solar radiation (kJ m^−2^ day^−1^), Prec = Mean annual precipitation (mm/year), Livestock = Livestock population (livestock population/5 arc minute), Vapr = water vapor pressure (kPa), Wind = wind speed (m s^−1^), Land cover = Land cover type.

A final ensemble model was created by incorporating weighted runs from the 21 models which met the inclusion criteria (all models using the RF, GAM, GBM, CTA, FDA and MARS algorithms, two using GLM and one using ANN). In this ensemble model, mean annual minimum temperature had the highest overall contribution (42.9%), followed by mean annual maximum temperature (21.1%), solar radiation (13.6%), annual precipitation (11%), livestock distribution (6.2%), vapor pressure (3.4%), wind speed (0.8%), and land cover (0.1%).

**Figure 2:**
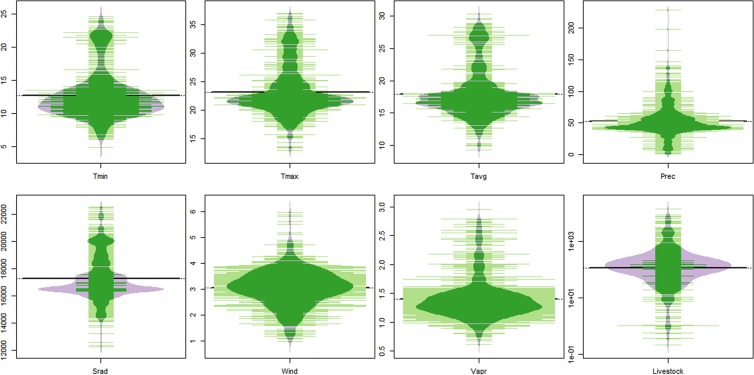
Beanplot of environmental and livestock demographic characteristics of *C*. *imicola* occurrence localities (N = 1039). Tmin = Mean annual minimum temperature (°C), Tmax = Mean annual maximum temperature (°C), Tavg = Mean annual temperature (°C), Prec = Mean annual precipitation (mm/year), Srad = Solar radiation (kJ m^−2^ day^−1^), Wind = wind speed (m s^−1^), Vapr = water vapor pressure (kPa), Livestock = Livestock population (livestock population/5 arc minute).

As indicated in Fig. 2, the presence localities have moderate temperature values. The mean annual minimum and maximum temperature of the occurrence localities were 12.7 ± 3.5 and 23.2 ± 4.2 °C, respectively. The annual precipitation of the occurrence localities was 52.7 ± 23.8 mm. The predominant land cover type of the occurrence localities is cultivated terrestrial areas and managed lands based on Land Cover Classification System (LCCS).

### Predicted geographical distribution of *C*. *imicola*

**Figure 3:**
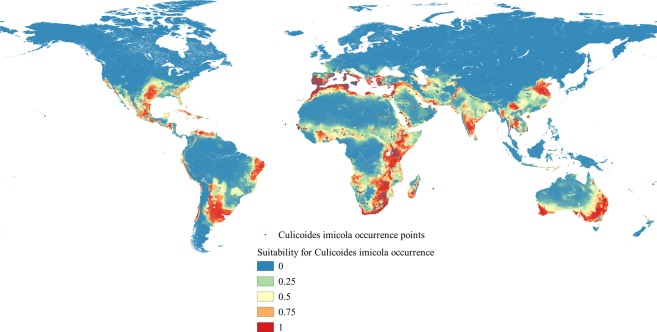
Predicted potential distribution of *C*. *imicola*. The scale indicates less suitable environment (cooler colors) and most suitable environment (warmer colors).

The ensemble model indicated high environmental suitability for *C*. *imicola* in the tropics and subtropics (Fig. 3). In Africa, the potential distribution of *C*. *imicola* was widely distributed across most Sub-Saharan African countries; except some central African countries, namely the Democratic Republic of Congo, Equatorial Guinea, Gabon and Republic of Congo. Habitat suitability for *C*. *imicola* was also predicted along the Mediterranean coast extending from Morocco to Egypt. Areas predicted to be highly suitable are found in southern, south eastern, and the Horn of Africa. In the Americas, the potential distributional of *C*. *imicola* was observed in many South American countries, particularly Brazil, Paraguay, Uruguay and Argentina. There were also environmentally suitable areas for *C*. *imicola* along the coasts of Venezuela and Columbia, extending to many Caribbean islands, and to much of the southern USA and Mexico.

In Asia, *C*. *imicola* potential suitability was indicated across south-east Asian countries including southern and eastern China, Myanmar, Thailand, Vietnam, and Cambodia. Potential suitability for the species also occurred in Syria, Lebanon, Israel, and along the south-western coasts of the Arabian Gulf. Considerable suitability for *C*. *imicola* was also observed in Iran, Iraq, Kuwait and the Indian subcontinent. In Australia and New Zealand, the predicted suitability for *C*. *imicola* included much of the south-western, eastern and southern coasts of Australia, and Northern New Zealand. In Europe, *C*. *imicola* was predicted to occur along the Mediterranean coasts of Turkey, Greece, Cyprus, Albania, Croatia, Italy, southern France, Spain and Portugal.

The model strongly matches most known occurrence patterns including the recent hotspots in southern Europe. It also predicts additional regions where *C*. *imicola* has so far been unrecorded, but where further inquiry may be warranted (in particular, southern USA and Mexico, the Caribbean and various South American countries). The model also indicates that certain occurrences, such as records from the Sahara Desert (north-western Sudan), are likely to be outside the stable niche.

### Model uncertainty

**Figure 4:**
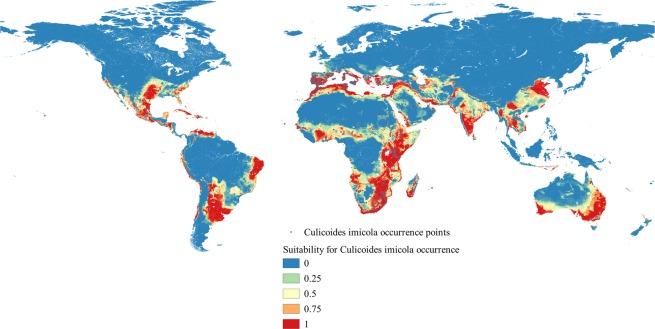
The estimated committee averaging across the selected predictions. The scale indicates unsuitable environment with certain prediction (cooler colors), less suitable with uncertain prediction (light colors), and most suitable environment with certain prediction (warmer colors).

**Figure 5:**
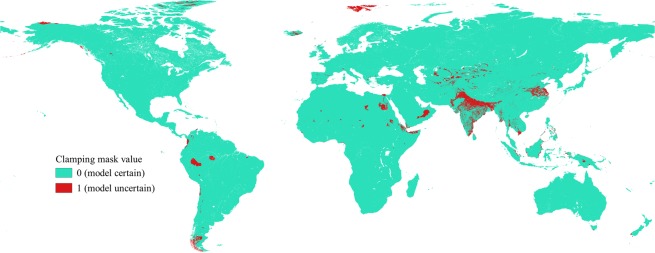
The Estimated ‘*clamping mask*’ value. Warmer colors indicate areas where models predictions are uncertain.

The committee averaging model depicted in Fig. 4 shows a prediction and a measure of uncertainty. When the prediction is close to 0 (blue) or 1 (red), it means that all models agrees to predict 0 and 1 respectively. When the prediction is around 0.5, it means that half the models predict 1 and the other half 0. Figure 5 shows the ‘*clamping mask*’ value. The values equal to one corresponds to uncertainty in models predictions. The model showed variation in the uncertainty index among different regions. Both the ‘*committee averaging*’ and ‘*clamping mask*’ value showed higher uncertainty in the Indian subcontinent. Uncertainty in models predictions was also observed for some part of Africa, central Asia, south eastern Asia and some part of Southern America. Thus, caution should be taken when interpreting the result for these areas.

## Discussion

The present study provided the most up-to-date and detailed maps of the predicted potential distribution of *C*. *imicola* using an ensemble modeling technique. The results presented here are based on thinned and unbiased treatment of the most extensive *C*. *imicola* occurrence dataset created to date, and represents the most up-to-date description of *C*. *imicola* distribution. To overcome the limitation of previous studies, we used additional sets of occurrence data^35^ and a robust modeling technique^22^. Our ensemble model performed well (TSS = 0.898 and ROC = 0.991), indicating a clear ability to distinguish between suitable and unsuitable habitat^41^.

Several factors may influence the distribution of *C*. *imicola*, including both biotic and abiotic factors. In this study, mean annual maximum temperature, mean annual minimum temperature, mean annual precipitation, solar radiation, water vapor pressure, wind speed and land cover type, which are abiotic factors, and livestock distribution which is a biotic factor, were required to model the distribution of the species. The global distribution of *C*. *imicola* appears to be limited by temperature; with annual minimum and maximum temperatures being the major determinants of the distribution of *C*. *imicola*. A study that aimed to map the potential distribution of *C*. *imicola* in Europe^16^, identified three temperature variables as significant determinants of the distribution of *C*. *imicola*, namely minimum of the monthly minimum temperatures, maximum of the monthly maximum temperatures, and the number of months per year with a mean temperature ≥12.5 °C. An experimental study by Veronesi *et al*.^45^ indicated that temperature could influence the fecundity, hatching and survival rate of *C*. *imicola*. In our ensemble model, temperature covariates contributed 64% to the model; the mean annual minimum and maximum temperature of the occurrence localities were 12.7 ± 3.5 and 23.2 ± 4.2 °C, respectively. According to Veronesi^45^, when reared at higher temperature (28 °C), *C*. *imicola* demonstrated higher variability in fecundity and lower hatching rates. On the other hand, the mean emergence rate from pupae was highest at 20 °C. However, distribution and abundance of *C*. *imicola* is likely directly constrained by their relatively poor tolerance of lower temperatures^46^. Other climatic factors, such as solar radiation and precipitation, were also important in determining the distribution of *C*. *imicola*.

Our models indicate that non-climatic factors are also important in driving spatial distribution of Culicoides including land cover and host factor (livestock distribution). The contribution of land cover to the model was low. According to Purse *et al*.^27^ local-scale abundance patterns of Culicoides were best explained by models combining host, landscape and climate factors. The land cover characteristics of *C*. *imicola* includes rainfed croplands, mosaic cropland (50–70%) / vegetation (grassland/shrubland/forest) (20–50%) and mosaic vegetation (grassland/shrubland/forest) (50–70%) / cropland (20–50%) based on LSSC land cover classification. In this study, livestock distribution was found to have an influence on the distribution of *C*. *imicola*. Evidence of the importance of livestock as sources of blood meals for *C*. *imicola* is well established^47^. *C*. *imicola* is a blood-sucking insect, tend to blood-feed on and breed near domestic livestock and humans. Frequencies of contact between Culicoides and vertebrate hosts are closely related to pathogen amplification and the risk for transmission^27,47^.

*C*. *imicola* is widely distributed across the world, from South Africa to the Mediterranean basin and across the Middle and Far East^4,35^. The suitability maps identified areas suitable for the occurrence of *C*. *imicola* in the tropical and subtropical regions. There are, however, areas with either low suitability or even completely unsuitable for *C*. *imicola* in this part of the world, underlining a patchy distribution of this species. As an example, although, tropical Africa is known to be within the global distribution range of *C*. *imicola*, the Sahara Desert and the heavily forested central Africa were found not to be suitable for this species. The suitability map indicates all tropical rainforests were unsuitable for the existence of *C*. *imicola*. *C*. *imicola* is a heliophile breeding predominantly in habitats that are low forest cover and open to sunlight^48^. It is important to note that, the suitability for *C*. *imicola* extends its known global distribution, as considerable suitable habitats are manifested in areas where no occurrence has so far been reported, including areas in southern USA, Mexico, Central and southern American countries, and Australia. Thus, it is entirely feasible that *C*. *imicola* could spread into these parts of the world, potentially increasing the risk of transmission of Bluetongue, and AHS.

A considerable number of studies have explored the role of different climatic factors on the distribution and abundance of *C*. *imicola*^16–18,20,24,45,46,48–58^. Climatic factors, mainly lower temperature limits may play a role in constraining a northern expansion of suitable habitat especially in Europe and Asia. As stated by Guichard *et al*.^4^, there appears to be substantial opportunity for range expansion of *C*. *imicola*. The model developed by Guichard and colleagues^4^ projects a northern expansion of suitable climate especially in Europe and some parts of Asia, under future climate scenarios.

The results of the current model overlap in many respects with the previously published model of Guichard and colleagues^4^. However, the current model resulted in different distributional patterns to those predicted by Guichard *et al*.^4^ in many parts of the world. For example, in contrast to the current model, the model proposed by Guichard and colleagues^4^ predicted wider suitability for *C*. *imicola* in Central Africa, the Pacific region, northern and western Brazil, and New Zealand. As such we postulate that the previous study^4^ over-estimated the potential distribution of this species. The distributional potential of the species was likely poorly estimated owing to the quality of the occurrence data, the quality and resolution of the predictor variables, as well as the predictive performance of the model used.

The distribution of BT and AHS overlaps with the predicted distribution of C. imicola. BT is a widely distributed disease with reports from Africa, Europe, and Asia. The distribution of AHS on the other hand is restricted to Africa and Middle East (OIE, 2009). Despite the presence of suitable habitats, and competent vectors, AHS didn’t introduce in to southern and Western Europe, Eastern Asia, Southern Asia, and South Eatern Asia. BT and AHS have a high potential to spread to other parts of the world via the transportation of infected livestock, or mosquitoes. Due to the presence of suitable habitats and a competent vector, the present study infers a considerable probability of BT and AHS introduction into countries of East and Southeast Asia and Oceania. European countries along the Mediterranean Sea have a significant risk of AHS virus introduction.

In the current study, a range of predictor variables were used. Climatic variables were obtained from WorldClim^32^. WorldClim has become a most widely used climatic data source when constructing species distribution models. Despite the extensive application of WorldClim data to species distribution modeling, the database is known to have some limitations^59^. WorldClim version 2 has average monthly climate data for minimum, mean, and maximum temperature and for precipitation only between 1970 and 2000, which may not accurately reflect current climatic conditions. Thus, the result displayed here may deviate from current reality in areas that have undergone clear climatic changes over the last two decades.

## Conclusion

This study has provided predictive estimates as to the potential distributions of *C*. *imicola* and could be used for decision making at global and regional scales. The model developed has importance for two main reasons; (1) the model estimate probabilities of occurrences for one of globally cosmopolitan and primary vectors of major arboviruses of veterinary importance that are increasingly spread across the world, and (2) the maps provide primary and firsthand information to prioritize surveillance and control programs for both *C*. *imicola* and the diseases transmitted by this species, such as Bluetongue, and AHS. The study enables the scientific community and policy makers to indirectly infer the risks of these diseases and thereby provides a framework for updating management and biosecurity strategies to target disease epizootics.

## Data Availability

The datasets generated during and/or analyzed during the current study are available from the corresponding author up on request.
